# Alveolar cells under mechanical stressed niche: critical contributors to pulmonary fibrosis

**DOI:** 10.1186/s10020-020-00223-w

**Published:** 2020-10-14

**Authors:** Juntang Yang, Xin Pan, Lan Wang, Guoying Yu

**Affiliations:** State Key Laboratory of Cell Differentiation and Regulation, Henan International Joint Laboratory of Pulmonary Fibrosis, Outstanding Overseas Scientists Center for Pulmonary Fibrosis of Henan Province, College of Life Science, Institute of Biomedical Science, Henan Normal University, Xinxiang, Henan China

**Keywords:** Pulmonary fibrosis, Mechanical stress niche, Alveolar cells

## Abstract

Pulmonary fibrosis arises from the repeated epithelial mild injuries and insufficient repair lead to over activation of fibroblasts and excessive deposition of extracellular matrix, which result in a mechanical stretched niche. However, increasing mechanical stress likely exists before the establishment of fibrosis since early micro injuries increase local vascular permeability and prompt cytoskeletal remodeling which alter cellular mechanical forces. It is noteworthy that COVID-19 patients with severe hypoxemia will receive mechanical ventilation as supportive treatment and subsequent pathology studies indicate lung fibrosis pattern. At advanced stages, mechanical stress originates mainly from the stiff matrix since boundaries between stiff and compliant parts of the tissue could generate mechanical stress. Therefore, mechanical stress has a significant role in the whole development process of pulmonary fibrosis. The alveoli are covered by abundant capillaries and function as the main gas exchange unit. Constantly subject to variety of damages, the alveolar epithelium injuries were recently recognized to play a vital role in the onset and development of idiopathic pulmonary fibrosis. In this review, we summarize the literature regarding the effects of mechanical stress on the fundamental cells constituting the alveoli in the process of pulmonary fibrosis, particularly on epithelial cells, capillary endothelial cells, fibroblasts, mast cells, macrophages and stem cells. Finally, we briefly review this issue from a more comprehensive perspective: the metabolic and epigenetic regulation.

## Background

Recent pathological studies reported some of the COVID-19 patients with lung fibrotic lung features. Clinically, the main consequence of SARS-CoV-2 infection is cytokine storm, however previous studies indicate that anti inflammation therapy have no effects on pulmonary fibrosis, thus other mechanisms need to be provided to address this issue. Early micro injuries to alveoli increase local vascular permeability which provokes edema accompanied by inflammatory cytokines which prompt cytoskeletal remodeling and alter cellular mechanical forces. If these mild injuries could not be repaired properly, then fibroblasts will be activated and subsequent excessive deposition of extracellular matrix will result in a mechanical stretched niche. At advanced stages, mechanical stress originates mainly from the stiff matrix since boundaries between stiff and compliant parts of the tissue could generate mechanical stress. Therefore, mechanical stress has a significant role in the whole development process of pulmonary fibrosis. The alveoli are covered by abundant capillaries and function as the main gas exchange unit. Constantly subject to variety of damages, the alveolar epithelium injuries were recently recognized to play a vital role in the onset and development of idiopathic pulmonary fibrosis. In this review, we summarize and the literature regarding the effects of mechanical stress on the fundamental cells constituting the alveoli in the process of pulmonary fibrosis, particularly on epithelial cells, capillary endothelial cells, fibroblasts, mast cells, macrophages and stem cells. Finally, we briefly review this issue from a more comprehensive perspective: the metabolic and epigenetic regulation.

## Main text

### Mechanical stretch- a critical player in pulmonary fibrosis

Pulmonary fibrosis (PF), which constitutes a broad range of heterogeneous end stage interstitial lung diseases, is characterized by excessive deposition of extracellular matrix and destruction of the pulmonary parenchyma (Lederer and Martinez 2018). Factors contributing to pulmonary fibrosis include genetic disorders, autoimmune diseases, occupational exposures, toxins, drugs, radiation and most recently the SARS-CoV-2 infection (Barratt et al. 2018; Chen 2020). Idiopathic pulmonary fibrosis (IPF) is the most aggressive form which may be pathologically indistinguishable from other forms, especially at the later stages (Raghu et al. 2011). IPF severely affects the respiratory function which is manifested by dry cough, and progressive dyspnea. The incidence and prevalence of IPF increase every year. It affects approximately 5 million people worldwide and reflects a significant health burden (Raghu et al. 2015). The median survival without lung transplant for IPF is almost 3 years, making it the non-lung cancer disease with the gravest prognosis (Sgalla et al. 2016). The initiation of IPF largely arises from the repeated epithelial injuries and insufficient repair leads to over activation of fibroblasts and subsequently fibrosis. Despite extensive research, the pathogenesis of IPF remains elusive (Sgalla 2018).

**Fig. 1:**
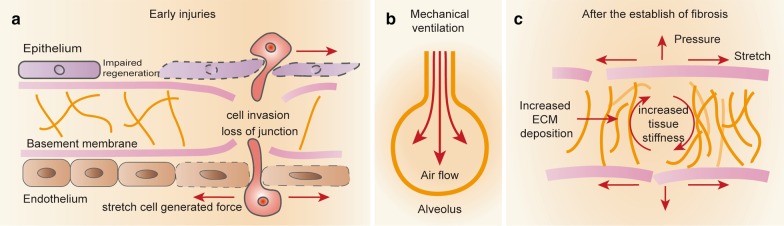
The three major sources of mechanical stress in pulmonary fibrosis. **a** Micro injuries in the initiation stage alter local epithelium and endothelium permeability and cell junction. **b** Mechanical ventilation induced alveoli stretch. **c** Excessive ECM deposition after the establishment of fibrosis forms a vicious cycle of pro-fibrotic state

Mechanical strain describes a condition when an object is deformed due to external factors such as the disruption of cell–cell conjunction (Tschumperlin et al. 2018). Mechanical stimulation plays an essential role for the physiological function. Particularly in the lung, mechanical stimulation within a physiological range provides the basis for maturation, tissue regeneration and functionality (Gomes et al. 2001; Schmitt et al. 2012) and it is widely believed that stretch of alveolar epithelial cells II, which occurs during breathing, is the predominant physiological trigger for surfactant release (Edwards 2001). However, mechanical stimulation above the physiological range (pathological stretch) is responsible for the development of lung injury and subsequent fibrotic response (Pugin 2003). In the process of lung fibrosis, the mechanical stress origin from three phases, at the initiation stage (micro injuries) (Fig. 1a); progression stage (moderate matrix deposition and supportive mechanical ventilation) (Fig. 1b) (Mooney et al. 2017) and advanced stage (Fig. 1c) [exaggerated and disordered deposition of extracellular matrix (ECM) and pulmonary artery hypertension (PAH)] (Hoffmann et al. 2014). Generally, after initial injury to the lung, activated epithelium cells release acute injury signals and recruit innate immune cells (Pellicoro et al. 2014; Wynn 2011) whereby vascular permeability increases which promotes the leakage of circulating fluid-phase components, therefore mechanical stress is induced by the disruption of endothelial–epithelial cells boundaries (Huse 2017). Ideally the micro injuries could be repaired, otherwise, this diffuse alveolar damage will lead to acute respiratory distress syndrome (ARDS) (one of devastating outcomes of SARS-CoV-2 infection) (Villar et al. 2019) and patients receive mechanical ventilation as a supportive treatment show great potential to develop lung fibrosis (Mooney et al. 2017; Mao et al. 2017; Cabrera-Benitez et al. 2012). If unfortunately fibrosis is established, tissue stiffness will inevitably increase (Tschumperlin et al. 2018; Wells 2013; Wu 2019) and a vicious cycle characterized by increasing in tissue stiffness via fibrillar collagen accumulation and cross-linking will be established (Jansen et al. 2013; Wen and Janmey 2013). Therefore, it is plausible to speculate that this rigid niche may interplay with local resident cells in the pathological process of PF.

We together with other groups defined the central role of alveolar epithelial cells injury in lung fibrosis (Yu et al. 2018a, b; King et al. 2011). The alveolar region (parenchyma) of the lung comprises about 90% of its total volume. The inter-alveolar septum provides the structural basis for gas exchange in the lung (Ben-Tal 2006). In general, five different functional cells constitute the structure of alveoli: epithelial cells (type I and type II), endothelial cells in the capillary lumen, fibroblasts in the interstitial tissue, macrophages and mast cells in the inner alveolar space (Knudsen and Ochs 2018). Under mechanical stressed situation, the behavior of these functional cells in the alveoli will be disturbed and largely determine the pathological process of lung fibrosis. Therefore, in order to illustrate the pathogenesis of pulmonary fibrosis from a previously overlooked perspective, we carefully review the studies regarding to the effects of mechanical stress on epithelial cells, endothelial cells, fibroblasts, macrophages, mast cells and alveolar stem cells in detail and briefly review this topic from a more comprehensive view: metabolic and epigenetic regulation.

#### Alveolar epithelial cell (AECs)

There are two types of epithelial cells in alveoli, type I and type II. Type I without proliferation ability is mainly responsible for gas exchange while type II is a progenitor cell with the potential to differentiate into type I after injuries and serve as the main source for surfactant production (Yu et al. 2019). Recent studies suggested that epithelial cell dysfunction played a vital role in the initiation of PF. Here we highlight the effects of mechanical stress on the alveolar epithelial cells in PF. The transcriptional cofactors YAP and TAZ are robust mechanosensors which translocate from the cytoplasm to the nucleus depending on matrix stiffness. Activation of YAP/TAZ could resolute inflammation via IκBa–NF-κB signaling, thereby accelerate alveolar epithelial regeneration and regression of fibrotic lesions (LaCanna et al. 2019; Lange et al. 2015), meanwhile MAPK-mediated YAP activation is essential for promoting alveolar regeneration in response to mechanical tension in the lung(Liu et al. 2016). Nevertheless, YAP can also activate mTOR/PI3K/AKT signaling to regulate abnormal cell proliferation, migration, and polarity in epithelial cells and contribute to the pathogenesis of pulmonary fibrosis (Gokey 2018). We think the distinct roles of YAP/TAZ activation in epithelial cells could reflect the fundamental mechanism of tissue fibrosis because fibrosis is the result of excessive or abnormal tissue repair. Since fibrosis mainly occurs as a consequence of failed tissue repair, the disruption of the AECs integrity acts as a trigger for fibrotic response (Rockey et al. 2015; Selman and Pardo 2006, 2014). Indeed, mechanical stress could induce oxidative damage and ER stress in AECs (Valentine et al. 2018; Lionetti et al. 2005; Tanaka et al. 2017) and lead to the releasing of injury molecules such as damage-associated molecular patterns (DAMPs) as initiation of tissue repair and fibrotic response (Wynn 2011; Ryter et al. 2018). The well-known DAMPs HMGB1 released by injured tissues promotes tissue repair by inducing migration and proliferation of stem cells, and by promoting angiogenesis (Yang et al. 2014; Tamai et al. 2011). However, HMGB1 could activate fibroblasts and promote endothelial cell proliferation, which might induce fibrosis as a program of tissue consolidation when successful regeneration is not achieved (Hamada et al. 2008; Zabini et al. 2015). If the injury continue exists, epithelial to mesenchymal transition (EMT), characterized by a series of events whereby epithelial cells lose their features, acquire mesenchymal properties and gain fibroblast-like phenotypes (Rout-Pitt et al. 2018; Kalluri and Neilson 2003), is necessary for proper re-epithelialization and repair (Stone et al. 2016). However, uncontrolled continuous EMT may result in fibrosis (Rout-Pitt et al. 2018; Hewlett et al. 2018; Chapman 2011; Kuwano 2007). Clinically, ARDS patients who receive mechanical ventilation are prone to develop lung fibrosis via EMT (Cabrera-Benitez et al. 2012) through the Midkine-Notch2-ACE signaling pathway (Zhang et al. 2015). Likely mir-19b overexpression promoted EMT in response to mechanical stretch through down-regulation of PTEN (Mao et al. 2017). These studies suggested that mechanical stress associated EMT contributed largely to lung fibrosis.

In normal alveoli, surfactant secretion is controlled by extracellular ATP and other nucleotides (Praetorius and Leipziger 2009), however, under pathological condition, overloaded ATP remains a major endogenous signal in promoting the release of pro-inflammatory cytokine IL-1β via activation of NLRP3 inflammasome through the binding of P2X7R receptor, thereby facilitating lung fibrosis progression (Gicquel et al. 2017; Riteau et al. 2010). Accordingly, increased ATP content was observed in the bronchoalveolar lavage fluid (BALF) from pulmonary fibrotic patients (Gicquel et al. 2017) and bleomycin induced lung fibrosis mice model (Riteau et al. 2010). Since a recent study showed that mechanical stretch stimulated the ATP release in lung alveolar cells (Grygorczyk et al. 2013), we hypothesize that the rigid fibrotic niche may form a vicious cycle which exacerbates lung fibrosis by ATP releasing. TGF-β is a widely recognized contributor of fibrosis and a recent study provided clues that under mechanical conditions, AECIIs may function as the source of TGF-β which can subsequently activate lung fibroblasts (Kuhn et al. 2019). In conclusion, under mechanical stress, not only AECs per se contribute to lung fibrosis but the molecules released by AECs also promote fibrotic response of adjacent cells.

#### Endothelial cell

The lung is a prominent place in the microvasculature with estimated capillary surface area (as defined by diameter of a vessel 10 µm or less) about 50–70 m^2^ (Weibel 1973), 20-times the surface area of all other vessels combined in the body (Wu and Birukov 2019). In the alveoli, the epithelium (type I) and endothelium form the “blood–gas barrier” for gas exchanging, therefore their stability mutually determine the homeostasis of lung (Knudsen and Ochs 2018).

Mechanical sensing and regulating of endothelial cells are involved in the process of lung disease (Fang et al. 2019). Endothelial mesenchymal transition (Endo-MT) depicts a scenario where endothelial cells acquire mesenchymal features to deposit extracellular matrix. Indeed, under stress condition, endothelial cells are implicated in the pathogenesis of tissue fibrosis. NLRP3 inflammasome activation contributes to mechanical stretch–induced Endo-MT and pulmonary fibrosis (Lv et al. 2018). A recent study showed that mechanosensing mediated endothelial cells metabolic change contributed to lung fibrosis via Endo-MT (Wu and Birukov 2019). Furthermore the rising stiffness of lung parenchyma increases the expression of TGF-β and HIF-1α in ECs which are necessary for the formation of PF (Kato et al. 2018; Bryant et al. 2016).The mechanical stress poses on endothelial cells exists not only in the early stages but also persist with disease progression. Clinically, pulmonary arterial hypertension (PAH) is featured by elevated mean pulmonary arterial pressure, pulmonary artery wedge pressure and pulmonary vascular resistance (Kovacs et al. 2018). The prevalence of PAH amongst PF patients is dependent upon the severity of PF. In the early stages or when initially diagnosed, PAH affects < 10% of patients (Klinger 2016). However, as disease advances, the incidence of PAH increases to 32% (Lettieri et al. 2006). Thus, PAH exposes the capillary endothelial cells to an extra mechanical stress niche which will further promotes the progression of lung fibrosis.

Taken together the mechanical stress exposed to the endothelial cells is translated to different signals for the initiation and progression of lung fibrosis.

#### Fibroblast

The migratory and proliferative fibroblasts organizing in distinct clusters is called fibroblastic foci and this depicts the typical phenotype of lung fibrosis featured by accumulation of exaggerated amounts of ECM that forms a stiff milieu (Liu et al. 2010; Pardo and Selman 2016; Zhou et al. 2013) where fibroblasts itself can sense the change of mechanical properties (Chen HP 2016; Coyer et al. 2012; Hinz 2012). Indeed, accumulating evidence suggests that fibrotic extracellular matrix provide a feed-forward mechanism that amplifies lung fibrosis (Hinz 2012; Rahaman et al. 2014; Wipff et al. 2007; Fiore et al. 2015; Tschumperlin 2013). The transcriptional cofactors YAP and TAZ are robust mechanosensors which translocate from the cytoplasm to the nucleus depending on matrix stiffness and number of studies show that YAP/TAZ are crucial for fibroblasts activation in responses to mechanical stress (matrix stiffness) which contributes to fibrotic features (Liu et al. 2015; Noguchi S 2017; Piersma and Bank 2017; Noguchi et al. 2018). α6-integrin senses the stiff matrix and confers an invasive phenotype of myofibroblast (Chen 2016) which contributing to the increased TGF-β in PF patients(Wipff et al. 2007). Previous study showed that S100 calcium binding protein A6 (S100A6) was up-regulated by mechanical strain in lung fibroblasts (Breen et al. 1999) and regulated the quiescent-activate transition of fibroblasts (Breen and Tang 2003). Functionally, S100A6 binds Ca^2+^ to interact with specific intracellular target proteins (Donato et al. 2017) and a recent study reported that Ca^2+^ influx can be induced by mechanical stretch in human lung fibroblasts (Murata et al. 2014). Thus, it can be postulated that mechanical stress facilitates the proliferation of lung fibroblasts through Ca^2+^ mediated S1006A stimulation. Taken together, these data indicate that mechanical stress promotes fibrosis by both stimulating the proliferation and migration of fibroblasts.

#### Monocyte/macrophage

Macrophages are involved in the cross-talk between innate and adaptive immunity (Epelman et al. 2014). Previously, they were thought to be solely derived from the circulating monocytes (Volkman and Gowans 1965), nonetheless recent studies indicated that most adult tissue-resident macrophages were colonized independently of circulating monocytes before birth (Epelman et al. 2014; Yona et al. 2013). The function and character of macrophage varies dramatically depending on their anatomical location such as the macrophages in alveolar space, adipose tissue, and liver (Wynn et al. 2013). The lung macrophages fall into two subgroups based the on their location: alveolar macrophages (AMs) residing in alveoli (Guilliams et al. 2013) and interstitial macrophages (IMs) staying in the parenchymal tissue (Bedoret et al. 2009). Beyond the AM/IM classification by localization, macrophages can shift dynamically between two activated forms: classically-activated (M1) and alternatively-activated (M2) in response to ever-changing environmental factors (Braune et al. 2017; Scott et al. 2014; Gordon and Martinez 2010). M1 macrophages mainly function in the host defense system to eliminate pathogens by generating pro-inflammatory chemokines and cytokines such as TNF-α, CCL2 and ILs (Saradna et al. 2018; Murray and Wynn 2011) whereas M2 harbors anti-inflammatory properties and engages in ECM remodeling (Mantovani et al. 2004). Despite the chemical factors, emerging evidence suggests that physical environment contributes to the regulation of macrophage polarization (Fereol et al. 2006; Pugin et al. 1998; McWhorter et al. 2015; Chu 2019; Shan 2019). As aforementioned, the PF lung exhibits a rigid condition by over deposition of ECM, thus it is reasonable to speculate the cross-talk between alveolar macrophages and the rigid alveolar environment. In the current paradigm of PF pathogenesis, sustained inflammatory responses would serve as a trigger to initiate and propagate fibrotic responses in lung (Zhang 2018). Indeed, mechanical stress induces synergistic pro-inflammatory effects of macrophages to release TNFα, IL-8 and IL-6 (Pugin et al. 1998). Similarly, stretch activates the NLRP3 inflammasomes and induces the release of IL-1β in mouse alveolar macrophages in a TLR4-dependent manner (Wu et al. 2013). These studies suggest that macrophages under stretched condition served as a contributor to PF. However, NO secreted by AMs via inducible nitric oxide synthase (iNOS) may function as part of a physiological anti-apoptotic mechanism to prevent AECIIs from undergoing stretch-induced cell death (Edwards et al. 2000), as we recently showed that AECIIs damage was key to the progression of IPF (Yu et al. 2018) thus macrophages after stretching in IPF may attenuate the vicious cycles of AECIIs injuries. Indeed Shan et al. showed mechanical stress induces the mouse murine Mφ RAW264.7 cells polarize to M1 via the FAK/NF-κB signaling pathway (Shan 2019), considering the deletion of TLR-4 (the major pathway for M1 macrophage activation) manifested increased susceptibility to bleomycin induced lung fibrosis (Jiang et al. 2005; Paun et al. 2010). Nevertheless, another group reported that mechanical stress polarized macrophage to M2 phenotype in hair regeneration (Chu 2019). This discrepancy could be partially explained as previously reported, a moderate strain was shown to increase the ratio of M2/M1 macrophages over a 7-day period (McWhorter et al. 2015),while a more extensive strain resulted in marked reduction in M2/M1 macrophages (Ballotta et al. 2014). In conclusion, the functions of macrophages in stretched fibrotic niche varies significantly depending both on the anatomical location and stretch intensity which reinforces the idea that an optimal or physiological level of stress promote tissue homeostasis, whereas abnormal ones leads to fibrotic response.

#### Mast cell

Mast cells, originate from CD34-expressing haematopoietic stem cells in the bone marrow, are best known for their roles in allergic and acute inflammatory diseases (Wernersson and Pejler 2014). However, an increasing number of studies revealed that abundant mast cells were observed in the human fibrotic lung tissue or in experimental pulmonary fibrosis models (Veerappan et al. 2013; Cha et al. 2012; Azuma et al. 2011). The association between mast cells and lung fibrosis remains a controversial topic regarding its pro-fibrotic (Hugle 2014; Wygrecka et al. 2013) or anti-fibrotic role (Bradding and Pejler 2018; Galli 2014). Mast cells, due to its function, contain secretory granules filled with various compound such as chymase, histamine and TGFβ (Wernersson and Pejler 2014) which are recognized as pro-fibrotic mediators (Overed-Sayer et al. 2014). In the late stage of fibrosis, the increasing mechanical force (Tschumperlin et al. 2018) promotes mast cell degranulation via RGD integrin dependent pathways (Fowlkes et al. 2013), and degranulation of mast cell is recently reported to activate TGF-β1 pathway in pulmonary fibrosis (Shimbori et al. 2019). Thus, we believe the discrepancy on the role of mast cells in lung fibrosis could be partially explained considering the stage of the disease. At the beginning, tissue resident mast cells sense injury and then initiate a synergetic action of injury repair naturally. When the damage is chronic or repetitive there will be of great potential for mast cells to betray and lead to tissue fibrosis by persistently releasing fibrotic mediators, especially in the stiffer fibrotic niche.

#### Stem cell

Histologically, PF develops from microscopic fibrotic areas at the very peripheral regions of lung and slowly progress inward (Plantier et al. 2011), which ultimately lead to respiratory failure (King et al. 2011) however, the mechanisms of this periphery-to-center progression remain unclear. Spatially, when lungs are inflated, the average distance between adjacent AECIIs at the periphery lung tend to be larger than that at the central lung (Wu 2019), which indicated a mechanical stressed situation. A most recent study showed that Cdc42^−/−^ AECIIs cannot regenerate new alveoli which resulted in sustained elevated mechanical tension that subsequently activates a TGF-β signaling loop in stem-like AECIIs and promote fibrosis (Wu 2019). Clinically IPF is recognized as an aging associated disease (King et al. 2011; Shanker S 2015; Mora et al. 2017). The lung grows progressively and reach peaks around of age 25 and after this peak, lung function declines with structural remodeling characterized by enlarged alveolar size which signifying more mechanical tension (Hecker 2018; Ochs et al. 2004; Schiller et al. 2019). This could partially explain why the aging population is more suspected to lung fibrosis from the perspective of mechanical stress.

#### Metabolism

Accumulating evidence suggests abnormal metabolism in PF (Rowan et al. 2019; Gaugg et al. 2019). However, studies on the mechanical stress induced metabolism dysfunction in pulmonary fibrosis are in their infancy. In particular, endothelial cells are exposed to a variety dimension of mechanical forces such as shear, press and stretch due to their special location (Wu and Birukov 2019). Indeed increasing evidence indicate that endothelial cell activation and metabolism associated transformation are controlled partially by mechanical forces (Fang et al. 2019; Sawada et al. 2014) and metabolic shift such as fatty acid oxidation has been related to the Endo-MT (Schoors et al. 2015; Xiong JH 2018) which is responsible for the origin of mesenchymal cell in lung fibrosis (Choi et al. 2016). Except for endothelial cells, static mechanical tension reduces the production of surfactant phospholipids in AECIIs which will in turn increases the mechanical tension of alveoli (Schmitt et al. 2012). Since mitochondria is the main organelle for respiration and lipid oxidation (Nunnari and Suomalainen 2012), future studies shedding light on the connection between mechanical stress and mitochondrial dysfunction will be of particular interest (Yang et al. 2020).

#### Epigenetics

The term “epigenetics” describes heritable changes in a cellular phenotype without alterations in the DNA sequence (Berger et al. 2009) and includes DNA methylation, histone modification, non-coding RNAs and chromatin remodeling (Berger et al. 2009; Dawson and Kouzarides 2012; Kontur and Giraldez 2017). Many biological and pathological processes are associated with epigenetic modifications and alterations, such as organogenesis, cancer, diabetes and fibrosis (Tzouvelekis and Kaminski 2015; Yang 2017; Yao et al. 2016; Zhang and Pollin 2018; Ikemori 2019). Among the factors implicated in the epigenetic regulation of pulmonary fibrosis (Helling and Yang 2015; Tzouvelekis and Kaminski 2015), the role of mechanical force is still emerging. Recently, evidence showed that the mechanical signal derived from stiffness matrix affected chromatin organization and global epigenetic state of the cells (Downing et al. 2013; Tan et al. 2014) and this may be associated with alteration of actin cytoskeleton and chromatin organization, which finally changed the accessibility for the transcription machinery (Le et al. 2016; Crowder et al. 2016). A recent study showed that MRTF-A controlled the deposition of H3K4 methylation on the promoters of pro-fibrotic genes and promoted their expression (Yu 2017; Fan et al. 2015). Beyond the DNA and chromatin, RNA methylation is another target of epigenetic modification (Sergiev et al. 2018). Until now no direct evidence regarding the link between mechanical stress and RNA methylation had been provided in lung fibrosis except a recent study showed that m^6^A modifications of pri-miRNA-126 was involved in the process of pulmonary fibrosis (Han 2019). What’s worse was that the mechanical stress information could be “memorized” via epigenetic mechanisms (Heo et al. 2015) which may have long-term effects on cell fate decisions (Yang et al. 2014). Indeed, a recent study revealed an important link between mechanical memory and lung fibrosis through miR-21 regulation (Li et al. 2017; Liu et al. 2010). Thus, uncovering the relation between rigid fibrotic environment and epigenetic alteration will be a promising field in lung fibrosis.

## Conclusions

**Fig. 2:**
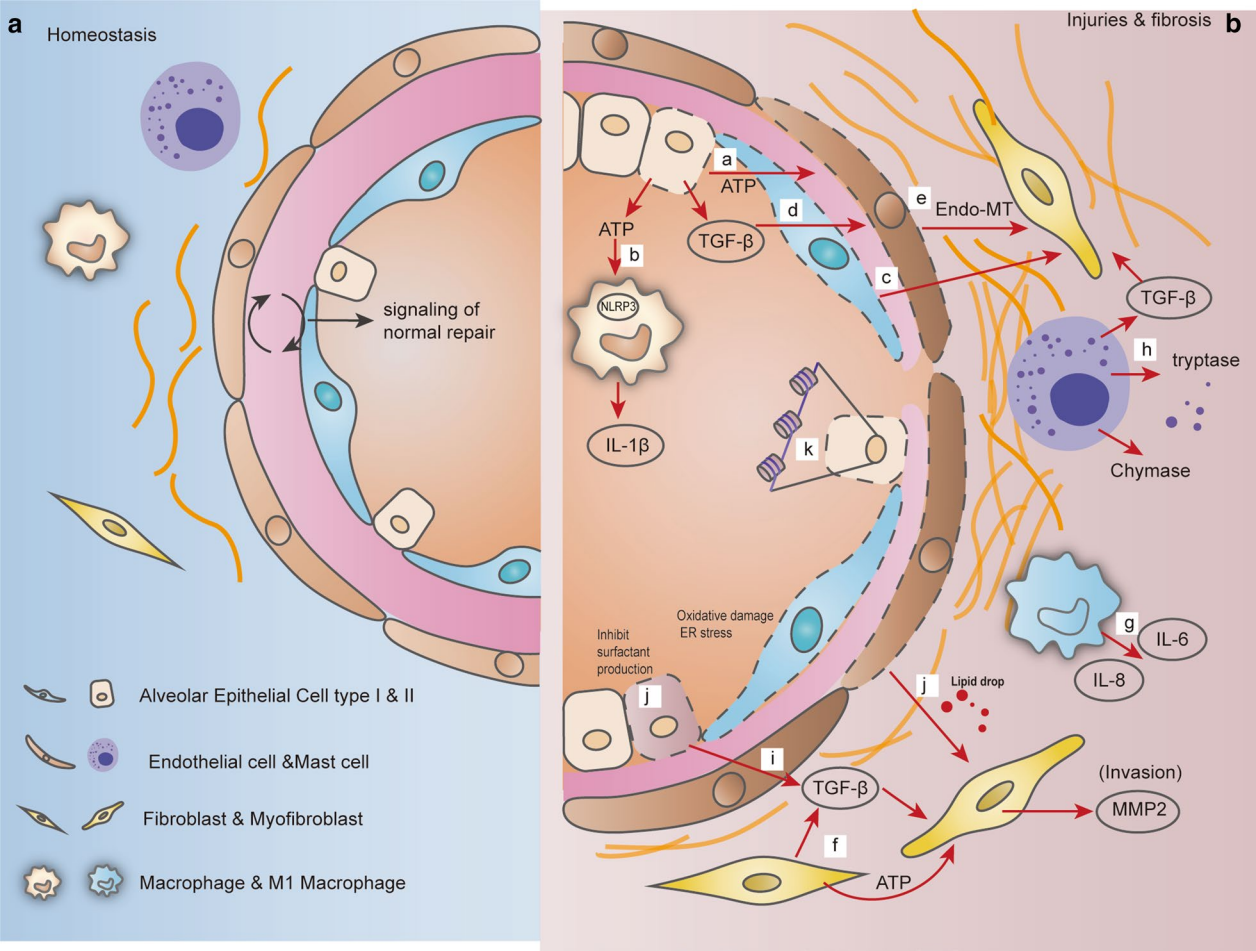
Rigid fibrotic niche accelerates the progression of lung fibrosis. **a** At homeostasis, communications between alveolar cells maintain the tissue integrity. **b** (a) Upon mechanical stress the ATP released by AECs promote Endo-MT of endothelial cells; (b) ATP also interact with P2X7R on macrophage to induce IL-1B production; (c) mtDNA released by AECs activate local fibroblasts; (d) TGF-β produced by AECs lead to the Endo-MT of endothelial cells and transformation of fibroblasts; (e) endothelial cells transform to mesenchymal cells via Endo-MT; (f) mechanical stressed niche contracts fibroblasts to activate TGF-β stored in the ECM and release ATP as pro-fibrotic mediator; (g) When subjected to mechanical stress machrophges will release IL-8 to activate mesenchymal progenitor cells (MPCs) and IL-6 to shifts acute inflammation into a more chronic pro-fibrotic state; (h) Mast cells response to mechanical stress by degranulation which subsequently release pro-fibrotic mediators such as tryptase, chymase and TGF-β to promote fibroblasts activation; (i) Alveolar stem cells -AECIIs under sustained elevated mechanical tension could liberate TGF-β stored in the ECM to promote fibroblasts activation; (j) mechanical stress affects the lipid metabolism in endothelial cells and contribute to Endo-MT, also static mechanical stress reduce the production of surfactant phospholipids in AECIIs; (k) Epigenetic regulation including DNA methylation, histone modification, non-coding RNAs and chromatin remodeling promote lung fibrosis by activating the transcription pro-fibrotic genes and miRNAs regulation and modification

In this review, we highlight the importance of rigid fibrotic niche in orchestrating the mechanical response of alveolar cells in exacerbating lung fibrosis (Fig. 2). The alveolar cells not merely respond to the mechanical stress by themselves, but also generate signal molecules to communicate with neighbor cells to promote fibrotic response. Upon mechanical stress the ATP released by AECs not only activate NLRP3 in endothelial cells to promote Endo-MT but also interact with P2X7R on macrophage to induce IL-1B production. As one kind of DAMPs, mtDNA is released by damaged AECs under mechanical stress and functions as activator of local fibroblast. Also TGF-β produced by AECs will lead to the Endo-MT of endothelial cells and transformation of fibroblasts. Mechanical stressed niche could contract fibroblasts to activate TGF-β stored in the ECM and release ATP as pro-fibrotic mediator. When subjected to mechanical stress, machrophges will release IL-8, which is able to activate mesenchymal progenitor cells (MPCs) and IL-6, which shifts acute inflammation into a more chronic pro-fibrotic state through induction of Th1 cell responses. Mast cells response to mechanical stress by degranulation which subsequently release pro-fibrotic mediators such as tryptase, chymase and TGF-β to promote fibroblasts activation. Alveolar stem cells -AECIIs under sustained elevated mechanical tension could liberate TGF-β stored in the ECM to promote fibroblasts activation.

Furthermore, mechanical stress exerts a more comprehensive impact via metabolic and epigenetic regulation, which should be a promising filed for treating PF since rapid advances have been made in developing drugs to adjust metabolic status and target the epigenetic landscape. Therefore, translating these mechanism insights into clinical utilization may enable novel approaches for alleviating lung fibrosis.

## Abbreviations

ACE: Angiotensin converting Enzyme
AECs: Alveolar Epithelial cell
AMs: Alveolar macrophages
ARDS: Acute respiratory distress syndrome
BALF: Bronchoalveolar lavage fluid
ECM: Extracellular matrix
EMT: Epithelial to mesenchymal transition
Endo-MT: Endothelial mesenchymal transition
PF: Pulmonary fibrosis
PH: Pulmonary hypertension
IMs: Interstitial macrophages
IPF: Idiopathic pulmonary fibrosis
